# Influence of aging on brain and web characteristics of an orb web spider

**DOI:** 10.1007/s10164-017-0530-z

**Published:** 2017-11-23

**Authors:** Alain Pasquet, Camille Toscani, Mylène Anotaux

**Affiliations:** 1 Faculté des Sciences et Techniques, University of Lorraine, UR AFPA, USC INRA n° 340, BP 239, Bld des Aiguillettes, 54506 Vandoeuvre-Les-Nancy, France; 2 University of Paris-Est, Ecole nationale vétérinaire d’Alfort, UMR 7179 CNRS MNHN, 94704 Maisons-Alfort, France; 3 CNRS, National Centre for Scientific Research, Paris, France

**Keywords:** Aging, Brain, Morphological parameters, Web construction, Orb web spider

## Abstract

In animals, it is known that age affects the abilities of the brain. In spiders, we showed that aging affects web characteristics due to behavioral alterations during web building. In this study, we investigated the effects of age on the associations between morphological changes to the spider brain and changes in web characteristics. The orb web spider *Zygiella x*-*notata* (Araneae, Araneidae) was used to test these relationships. Experiments were conducted on young (19 ± 2 days after adult molt, *N* = 13) and old (146 ± 32 days, *N* = 20) virgin females. The brain volume decreased with age (by 10%). Age also had an impact on the number of anomalies in the capture area generated during web building. The statistical relationships between the volume of the brain and web characteristics showed that there was an effect of age on both. Our results showed that in spiders, aging affects the brain volume and correlates with characteristics (anomalies) of the web. As web building is the result of complex behavioral processes, we suggest that aging affects spider behavior by causing some brain alterations.

## Introduction

In vertebrates, aging is associated with a decrease in motor and cognitive abilities. This can lead to important diseases such as schizophrenia, Huntington’s, Parkinson’s, and Alzheimer’s (Best and Alderton 2008). It has now been shown that these alterations are strongly correlated with specific morphological and anatomical features of the brain (Burke and Barnes 2006). As a consequence, the deterioration with age of some behavioral traits could be used as an indicator of the neurobiological degradation of the brain. This link is now formally established, but we lack animal models on which to test it. A solid model would be useful for elucidating the influence of aging on animal physiological and behavioral traits, as it would facilitate studies of the association between brain and behavior. Although a number of interesting animal models to study aging have been proposed (Keller and Murtha 2004; Ricklefs 2010; Edrey et al. 2011), there is not enough diversity to study the effect of aging on the link between brain and behavior (Carey et al. 2006).

Aging may affect animal behavior, and losses of behavioral performance could be linked to modifications to the central nervous system. A loss of neurons or a reduction in synaptic connections may underlie the relationship between behavior and the brain (Gallagher 1997; Lacreuse and Herndon 2009). In invertebrates, aging may affect the properties of neurons, their morphology, and also their connections through synapses (Williams et al. 2000). These alterations may lead to the loss of neurons or poor functioning (Yeoman and Faragher 2001). Neurons are directly associated with the secretion of neurohormones or neurotransmitters that circulate in the neuronal structures and provide the basis for brain regulation; these phenomena could be involved in the process of longevity in animals (Suo et al. 2009). Neurohormone regulation is one of the keys to the expression of behavior. These substances may regulate the behavior of several invertebrates: they modify *Drosophila* mating behavior (Certel et al. 2007), lobster locomotion (Tierney et al. 2004), the division of tasks among bees (Schulz and Robinson 2001), and even antipredator behavior in spiders (Jones et al. 2011).

Orb webs appear to be a good model in which to study the relationships between behavioral and brain changes with age. The orb web is the result of coordinated and stereotyped behaviors, and anomalies in the structure of the web result from behavioral errors during web building (Eberhard 2010, 2011; Toscani et al. 2012); these behavioral errors also increase with aging (Anotaux et al. 2012, 2015). In general, orb web design can vary due to environmental or internal factors (Herberstein 2011; Eberhard and Hesselberg 2012). Spiders modify the structure of their web when exposed to drugs or pesticides (Witt et al. 1968; Hesselberg and Vollrath 2004; Benamú et al. 2013; Pasquet et al. 2016). These variations are the result of behavioral adaptations. Anomalies in the otherwise perfect design of the orb were recently described (Pasquet et al. 2013). Thus, it is relevant to investigate them given that they reflect behavioral changes.

The spider brain is situated in the cephalic part of the cephalothorax. In spiders, as in most other arthropods, the central brain is a compact block divided in two parts: one situated above the esophagus near the venom glands and called the supraesophageal ganglion, and one situated below the esophagus in a ventral position and called the subesophageal ganglion (Barth 2002; Foelix 2011; Hesselberg 2010). The supraesophageal ganglion is connected to the sensorial and mechanical appendices of the cephalothorax (eyes, mouth parts, venom glands, and pedipalps) and is a nervous centre for cognitive functions (Barth 2002; Herberstein 2011). The subesophageal ganglion is connected to the spider’s legs and is more of a motor nervous centre (Barth 2002; Herberstein 2011). The size of the nervous system varies with species and developmental stage (Eberhard 2010). Some studies have shown that smaller spiders have a larger brain relative to their size (Quesada et al. 2011), but this was not associated with the behavioral abilities of the species. The same phenomenon was observed when comparing different stages of a given species. For example, in juvenile spiders of the genus *Mysmena*, which have body masses of <0.005 mg, the brain occupies 63% of the volume of the cephalothorax, but this proportion is only 48% for females (Quesada et al. 2011). Thus, in spiders, the brain is concentrated in the cephalic part of the body, and is easy to identify and remove.

In the present study, we investigated the relationship between aging, the brain, and behavior, using web building as an indicator of the behavior of the spider *Zygiella x*-*notata*. Our hypothesis was that morphological changes to the brain would be associated with a lack of motor coordination during web construction, leading to the presence of structural anomalies in the geometry of the web.

## Materials and methods

### Spider species

*Zygiella x*-*notata* is a medium-sized (5–7 mm for adult females) orb web spider that is widespread in northern Europe and establishes preferentially in the vicinity of human buildings (Roberts 1995). It constructs an orb web, which is generally characterized by the presence of a free sector in the upper part, and feeds primarily on flying prey (generally dipterans). In eastern France, its development cycle is annual: the juveniles leave the egg sacs at the beginning of spring; reproduction starts in summer with mating, and females lay eggs in September–October (Roberts 1995). As adults, the female life span is approximately 4–6 months (from August to December), but some individuals may survive until the following spring.

### Web building and web parameters

We compared two sets of spiders: young adult females (*N* = 13) and old females (*N* = 20). All females were captured in the field as subadults in August and brought back to the rearing room with a temperature of 19 °C and a 12 h/12 h daylight cycle (light from 8 a.m. to 8 p.m.). Females molted in the lab and they were all virgin. The older females were tested from 122 to 237 days after their molt and the young ones between 2 and 3 weeks after molting. All the spiders were weighed before the experiments, and the total length of the first forward leg was measured after the experiment. For the building tests, all spiders were put in large wooden frames (50 × 50 × 10 cm) enclosed by two windowpanes.

The building test lasted 5 days; after that, spiders that had not built a web were placed back in their boxes and removed from the study. The presence of a web was checked for every day. As soon as a spider completed a web, the frame was opened and web parameters were directly measured using electronic calipers. Direct measurements were made on the photographs following the method of Venner et al. (2001); measurements of the vertical and horizontal inner and outer radii were taken, and the number of spiral loops in the four directions was counted. From these measurements, we estimated the spider’s investment in the web by calculating the total length of the capture spiral (capture thread length (CTL), following Venner et al. 2001). The webs were photographed (Lumix FZ18) by placing them in front of a black panel using artificial light. Anomalies (as defined in Pasquet et al. 2013) in web construction were identified on photos and counted. Anomalies can affect the radii or capture spiral. For radii, we counted the number of supernumerary, deviated, and “Y” radii (Pasquet et al. 2013). For the capture spiral, we took into account the holes, the silk threads of the capture spiral stuck, and the number of nonparallel and discontinuous silk threads (Pasquet et al. 2013). For analysis, we used two parameters: the total number of anomalies (we added the number of radius anomalies to the number of capture spiral anomalies) and the number of capture spiral anomalies per cm of spiral length.

### Brain extraction and morphology

**Fig. 1:**
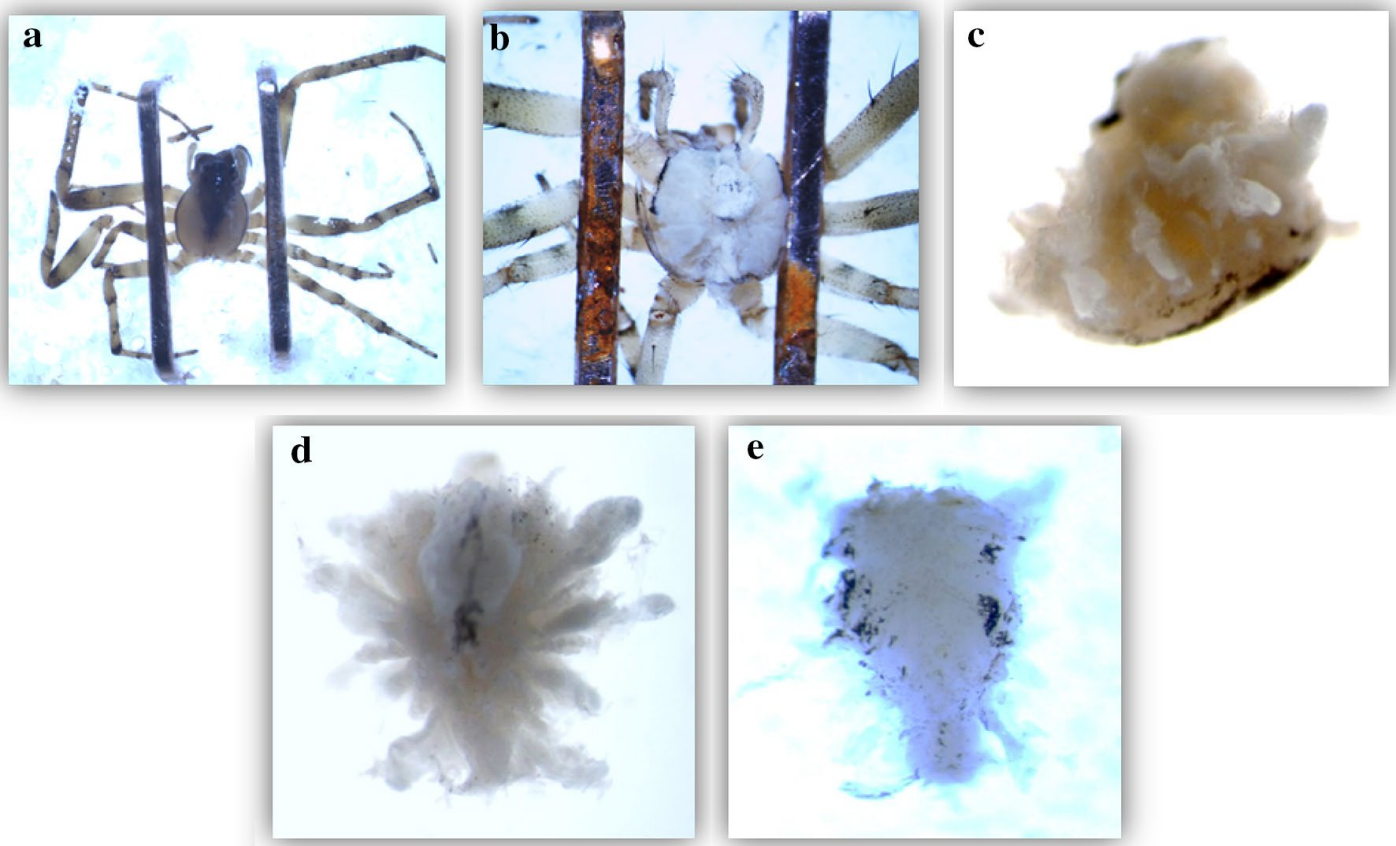
Dissection of the brain of an adult female of *Zygiella x*-*notata* (a young 21-day-old female): **a** immobilization of the cephalothorax; **b** the dorsal part of the cephalothorax cuticle was removed; **c** the brain (view of the lateral face) was completely extracted by removing all the fatty mass and the apodemes; **d** view of the dorsal side and **e** of the central region (pictures: M. Anotaux)

**Fig. 2:**
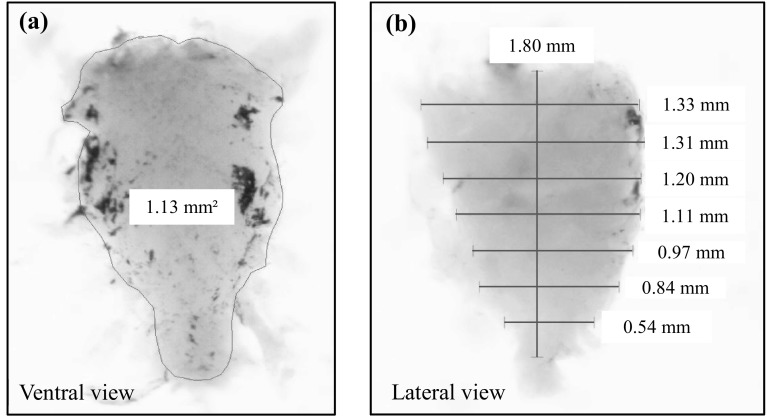
The measurements taken of each brain in two different planes: we measured the surface of the brain in the ventral plane (**a**),and we measured seven distances in the lateral plane (**b**) (see “Materials and methods”). We used the mean of these distances to calculate the brain volume (pictures: M. Anotaux)

After web building, to study brain morphology, we first placed the spiders into a freezer (− 80 °C) for 10 min and then into alcohol (70%) for 4 days. The brain was extracted from the cephalothorax, which was secured in a Petri dish on paraffin with staples in a 70% alcohol solution (Fig. 1a). This was positioned under a binocular microscope fitted with a camera (Optika binocular microscope, Sony camera). Photos were taken during the extraction. First we separated the abdomen and the cephalothorax, and then the dorsal part of the cephalothorax was removed so that the organs (the venom glands, the esophagus, and the brain) were visible. The brain is connected to the other structures by apodemes. These were cut and the total brain was extracted from the cephalothorax (Fig. 1c–e). The brain was measured in two positions; the total surface of the brain was measured viewing from the dorsal side (Fig. 2a; Archimed software), and then it was rotated 90° to obtain a lateral view. On this side, we divided the brain into eight equal parts along its length to give seven internal width measures, and we calculated the average to obtain the mean brain height (*H*_brain_) (Fig. 2b). These two measurements—the surface (*S*_brain_) and the mean brain height (*H*_brain_)—were multiplied to obtain the brain volume using the formula

$$V_{\text{brain}} = S_{\text{brain}} \times H_{\text{brain}} .$$

### Data analysis

We first examined the normality of the data with a Shapiro test and the homogeneity of variance with a Levene test. When the requisite conditions were fulfilled, we used parametric tests; nonparametric tests were used when one condition was not fulfilled.

We used a Student’s *t* test or a Mann–Whitney test (*U*) to compare brain morphological parameters and web characteristics between the two groups of young and old spiders (Table 1).

**Table 1:** Mean (± standard error) values of the parameters for the spiders and the web characteristics

	Old spiders (*N* = 20)	Young spiders (*N* = 13)	Statistical test	*p* value
Age (days)	146 ± 32	19 ± 2	–	–
Spider weight (mg)	39 ± 32	29 ± 12	*U* = 77	0.05
Leg length (mm)	10.95 ± 1.24	10.76 ± 1.73	*t* test = 0.36	NS
Brain volume (mm^3^)	1.18 ± 0.26	1.31 ± 0.28	*t* test = 1.35	NS
Length of spiral thread (cm)	587 ± 284	570 ± 197	*t* test = 0.26	NS
Capture area (cm^2^)	177 ± 80	168 ± 75	*t* test = 0.33	NS
Total anomalies	63 ± 26	46 ± 17	*t* test = 2.14	0.04
Anomalies/cm CTL	0.10 ± 0.05	0.07 ± 0.03	*t* test = 3.06	0.005

Different statistical tests were used depending on the normality of the data (*t* test = Student’s test and *U* = Mann–Whitney test)

A series of general linear models were used for the analyses (R: lmer, package ‘lme4’): first with age as a fixed effect, spider weight as a random effect, and brain volume as the dependent variable (model = lmer(brain volume ~ (age + weight))); second with age as a fixed effect, spider weight as a random effect, and number of anomalies of the capture spiral as the dependent variable (model = lmer(number of anomalies ~ (age + weight))). In each analysis, residuals were tested for homogeneity to validate the model. When the model was validated, a correlation table for various statistical models was generated in order to calculate *F* tests (R: Anova, package ‘car’), followed by a simultaneous test for general linear hypotheses as a post hoc test with Bonferroni correction (R: glht, package ‘multcomp’). As we demonstrated in a previous study that the length of the spiral thread (CTL) can be affected by age (Anotaux et al. 2012), all other web characteristics were statistically corrected for the CTL by using it as covariable in a linear regression model. Statistical analyses were carried out with the R package (version 2.15.0). *p* < 0.05 was considered to indicate statistical significance.

## Results

### Spider characteristics and relationships with web characteristics

Spider mass differed between young and old spiders (*U* = 77, *df* = 31, *p* = 0.05, Table 1), but the length of the first forward leg did not differ between those groups (*t* test, *t* = 0.36, *df* = 31, *p* > 0.10, Table 1). There were relationships between spider body characteristics and web characteristics: the number of anomalies per cm of the spiral thread tended to increase with spider mass (SE (standard error) = 6.8×10^−4^, *t* = 1.72, *df* = 31, *p* = 0.08) and length of the first leg (SE = 6.6×10^−3^, *t* = 1.95, *df* = 31, *p* = 0.06). The length of the capture spiral thread and the capture area did not vary with spider body mass (length of capture spiral thread: SE = 3.9, *t* = 0.33, *df* = 31, *p* = 0.74; capture area: SE = 1.1, *t* = 0.23, *df* = 30, *p* = 0.82), but they increased with the length of the first leg (length of capture spiral thread: SE = 39.1, *t* = 2.03, *df* = 31, *p* = 0.05; capture area: SE = 10.4, *t* = 2.83, *df* = 30, *p* = 0.008).

### Brain volume and web characteristics

**Fig. 3:**
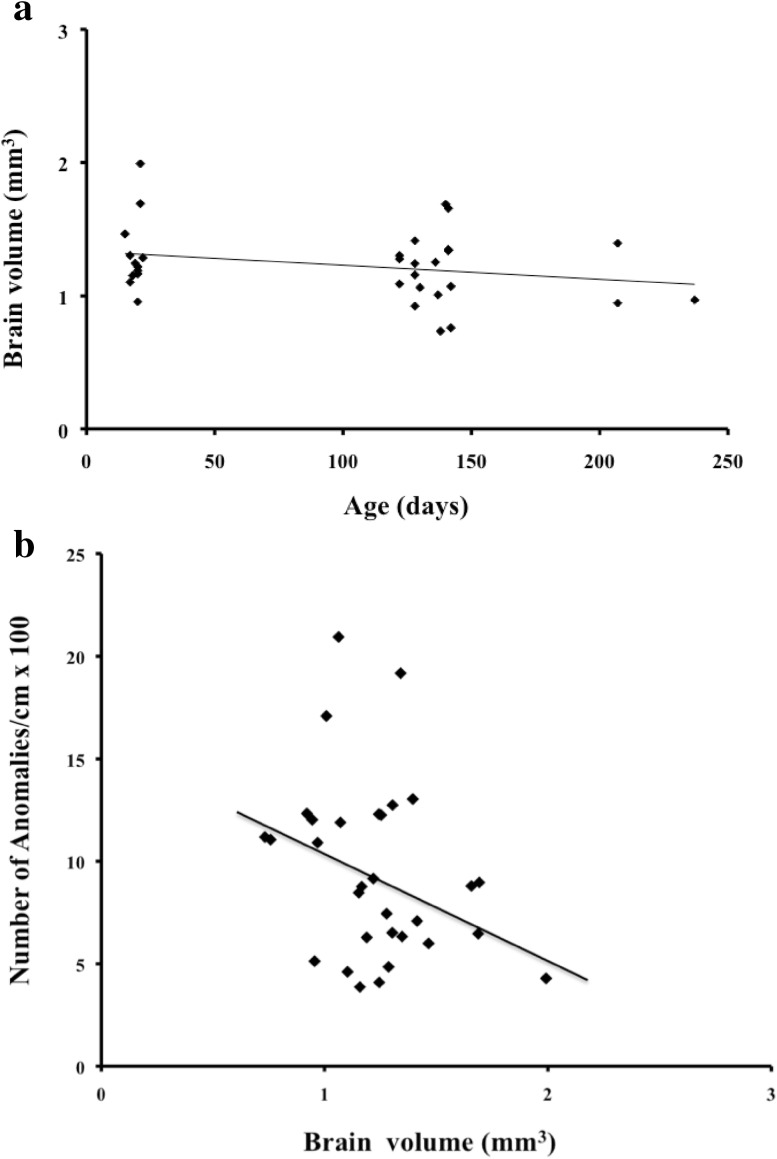
Relationships of brain volume with **a** spider age (*y* = 1.34 − 0.001*x*, *r* ^2^ = 0.07) and **b** the number of anomalies per cm of the final spiral thread (CTL) (*y* = 15.6 − 4.9*x*, *r* ^2^ = 0.10)

Brain volume did not differ between the two groups (*t* test, *t* = 1.35, *df* = 30, *p* = 0.19, Table 1). Adjusted for body mass, brain volume decreased with age (SE = 6.00×10^−4^, *t* = 2.62, *df* = 31, *p* = 0.03, Fig. 3a). Some web characteristics did not change with age; the length of the capture spiral (CTL) (*t* test, *t* = 0.26, *df* = 31, *p* > 0.10, Table 1) and the capture area (*t* test, *t* = 0.33, *df* = 31, *p* > 0.10, Table 1) were not significantly different for webs built by young or old spiders. The total number of anomalies in the web was higher for old spiders than for young ones (*t* test, *t* = 2.14, *df* = 31, *p* = 0.04, Table 1). The number of anomalies in the capture area per cm was more pronounced in the webs built by old spiders than in those built by young ones (*t* test, *t* = 3.06, *df* = 31, *p* = 0.005, Table 1).

### Relationship between brain volume and web characteristics

When we adjusted the brain and web characteristics for spider mass, the number of anomalies per cm of the capture area decreased with brain volume (SE = 0.03, *t* = 2.12, *df* = 31, *p* = 0.04, Fig. 3b). On the other hand, the length of the capture spiral, the capture area, and the number of radius anomalies did not change with brain volume (length of the spiral thread: SE = 202.2, *t* = 0.67, *df* = 31, *p* = 0.50; capture area: SE = 56.8, *t* = 1.34, *df* = 31, *p* = 0.19).

## Discussion

Our results showed that the volume of the brain decreased with age. The brain is an association of different cell types (Barth 2002) which play different roles in its functioning, and the reduced volume with age may be interpreted as losses of these different types of cells. The first interpretation is that there were fewer cells in the older spider brains compared to the younger brains. The second interpretation is a methodological one: all brains were extracted under water, and some tissues could have been damaged during the dissection. Thus, there was a possible loss of biological material during the brain dissection, but there is no reason that this should impact young spiders differently from old spiders.

When orb web spiders build their webs, different anomalies can occur in the structure of the web, and these anomalies are associated with different behavioral steps during construction. Some substances (chemical products, drugs, medicine) can affect many elements of the web (spiral loops, radii), but in most cases the final structure looks like an orb web (Witt et al. 1968; Reed et al. 1965; Hesselberg and Vollrath 2004; Benamú et al. 2010). In this study, the majority of the anomalies were structural faults that did not significantly affect the overall design of the orb web. Anomalies in web structure are often seen for orb web spiders (Pasquet et al. 2014): they can affect the capture spiral as well as the radii. Some (i.e., spiral turns that are stuck together and a discontinuity between two elements of the capture spiral) are the consequence of behavioral errors (Toscani et al. 2012), but we also previously showed that these anomalies could be due to spider age (Anotaux et al. 2012, 2015). Here, the older spiders made more anomalies during web construction than the younger ones. We previously suggested that this difference could be due to age-related modifications to the nervous central system. Age may decrease brain function, leading to errors during web construction. The influence of age on neuronal structures is known in invertebrates (Yeoman and Faragher 2001), but few studies have examined its effects on behavior.

The size limitation hypothesis predicts that small animals with smaller brains (and fewer neurons) are limited in their behavioral abilities. However, in spiders, various studies focusing on brain size and the complexity of behaviors involved in constructing an orb web do not agree with this hypothesis. Smaller spiders with smaller brains were able to build the same web without any structural differences (Eberhard 2007; Hesselberg 2010; Eberhard and Wcislo 2011). Furthermore, there was no difference between the orb webs built by juveniles and those built by adults of the orb-weaving spiders *Eustala illicita* and *Nephila clavipes* (Hesselberg 2013). The relationship between spider brain size and body size is not linear; the size of the brain increases less than the size of the body during development, and the size of the spider brain is relatively large for a small animal (Beutel and Haas 1998; Seid et al. 2011; Striedter 2005; Wehner et al. 2007). Some other factors may also influence the web characteristics during construction, such as spider mass (Venner et al. 2003), which increases with age.

The loss of physical abilities with age is a well-known phenomenon in long-lived animals such as mammals or birds, but it also occurs in short-lived species such as most invertebrates (Ridgel et al. 2003; Grotewiel et al. 2005; Murakami and Murakami 2005; Ridgel and Ritzmann 2005; Lliadi and Boulianne 2010), including spiders (Moya-Laraño 2002; Anotaux et al. 2014). This reduced physical ability may not be directly linked to a loss of neuronal performance. Here we did not test the abilities of the spiders to capture and eat prey, but some of the web characteristics that were modified could influence prey interception and retention. The link with the brain is complex because it is known that the different structures of the central nervous system play different roles. The spider brain consists of two functional parts: the supraesophageal ganglion, which is considered a nervous centre for cognitive functions, and the subesophageal ganglion, which is considered to be more of a motor nervous centre (Barth 2002; Herberstein 2011). Both ganglia are involved in web construction, because this activity requires cognitive performance, sensitive connections, and motor coordination (Eberhard and Wcislo 2011). Thus, a decrease in any brain structure could have a significant effect on the overall building behavior of orb web spiders.

## Conclusion

Our study clearly established that age has an impact on spider brain structures and behavior. These results show that age affects the brain and behavior of spiders in the same way, and that brain volume and behavioral performance decreases with age. This is a well-known phenomenon, even in invertebrates, but (to our knowledge) this is the first time that it has been observed in spiders.
